# Supplementary material for: Dandelion (Taraxacum mongolicum) Extract Alleviated H2O2-Induced Oxidative Damage: The Underlying Mechanism Revealed by Metabolomics and Lipidomics
Source: Foods. 2023 Sep 3;12(17):3314. doi: 10.3390/foods12173314 (PMC10486514; doi:10.3390/foods12173314)
Supplement: Supplementary file 1 [file foods-12-03314-s001.zip › foods-2578508-supplementary.pdf]

Dandelion (*Taraxacum mongolicum*) Extract Alleviated H_2O_2-Induced Oxidative Damage: The Underlying Mechanism Revealed by Metabolomics and Lipidomics

Yannan Chen [1,2], Siyuan Fei [1,2], Xiaoting Yu [1,2], Mingqian Tan [1,2,*]

[1] Academy of Food Interdisciplinary Science, School of Food Science and Technology, Dalian Polytechnic University, Qinggongyuan1, Gangjingzi District, Dalian 116034, China; ynchen04@163.com (Y.C.); 13470319363@163.com (S.F.); xiaoting12ming@126.com (X.Y.)

[2] National Engineering Research Center of Seafood, Dalian Polytechnic University, Dalian 116034, China

* Corresponding author (M. Tan, mqtan@dlpu.edu.cn).

Negative (-)

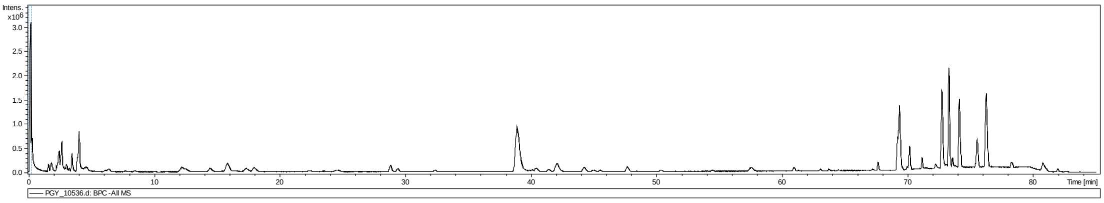

Positive (+)

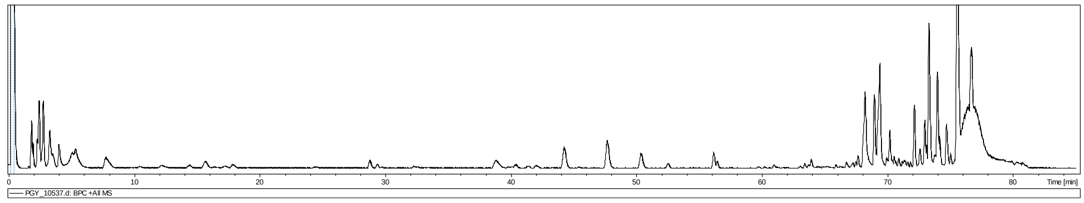

Figure S1. Total ion chromatogram of mass spectrometer of DE.

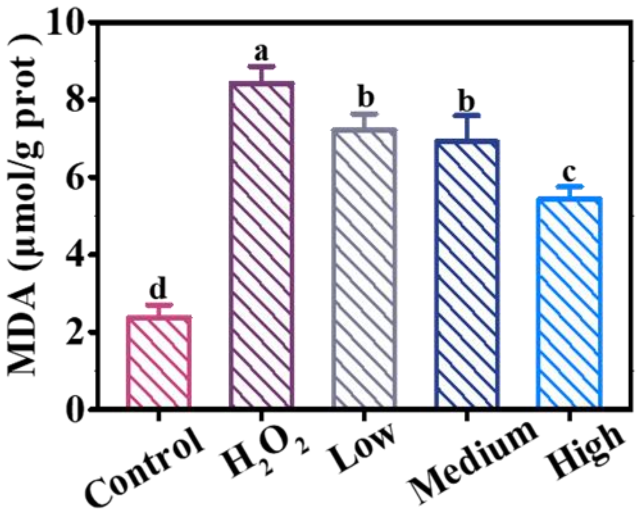

Figure S2. The level of MDA in different treated groups. Different letters indicated significant difference at $p<0.05$.

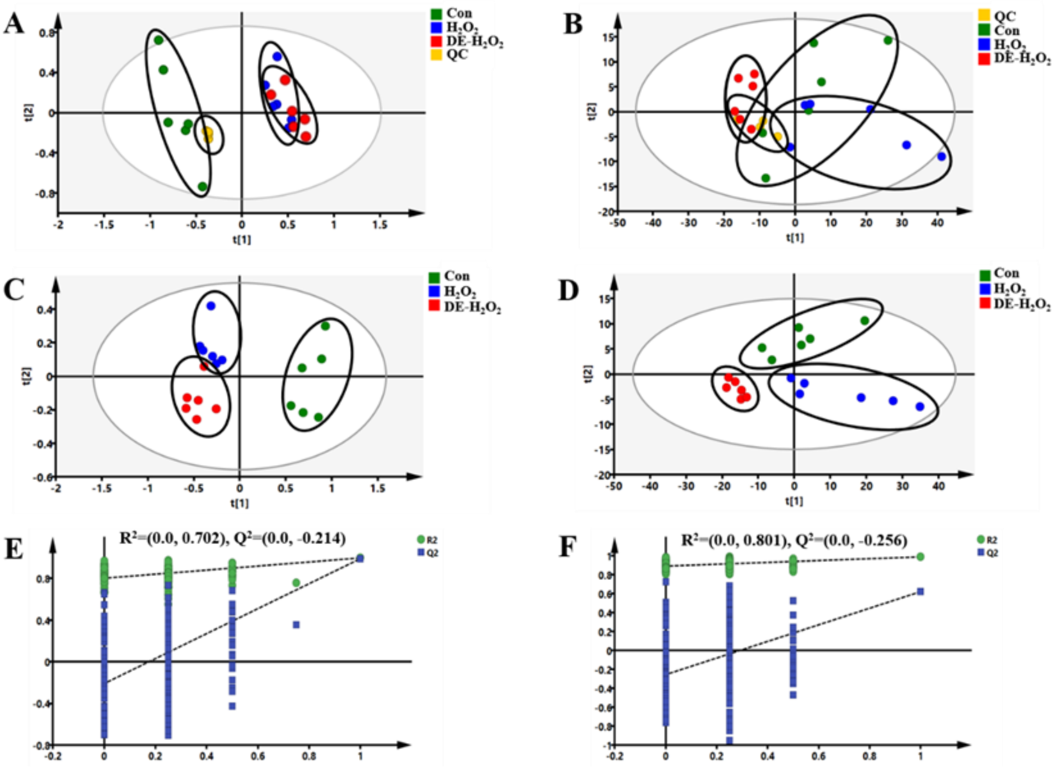

Figure S3. The metabolic profiles of PC12 cells with different treatment. PCA score plots for (A) metabo-lomics and and (B) lipidomics, PLS-DA score plots for (C) metabolomics for (D) lipidomics, a total of 200 random permutation tests of the PLS-DA model for (E) metabolomics and (F) lipidomics.

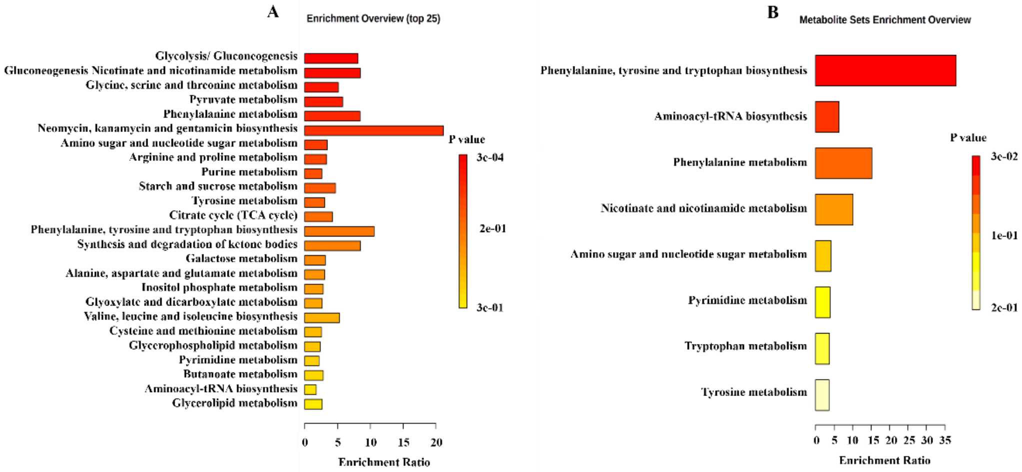

Figure S4. Enrichment analysis of differential metabolites in PC12 cells under different treatment. (A) the effects of H_2O_2 on metabolic pathways in comparation with the control, (B) the effects of DE-H_2O_2 on metabolic pathways in comparation with the control

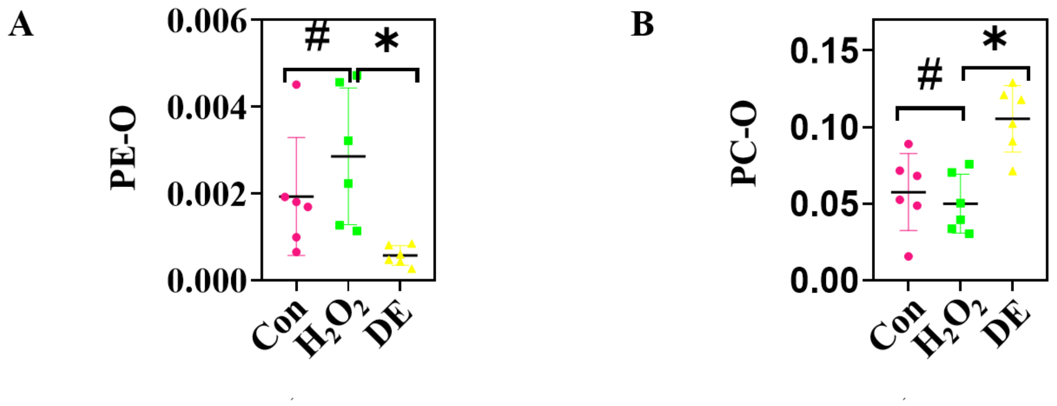

Figure S5. The relative level of PE-O (A) and PC-O (B) in PC12 cells after being treated with H2O2 or H2O2 in the presence of DE. "*" means significant differences (p < 0.05). "#" means significant differences (p < 0.01).

Table S1. Primer sequences used for RT-PCR.

Genes	Forward primer	Reverse primer
SOD1	TCGTCTTGCTCTCTCTGGTCC	CTGGTTCACCGCTTGCCTTC
GSH-Px	GACACCAGAATGGCAAGAATGAA	GACACCAGAATGGCAAGAATGAA
GAPDH	CCTCGTCCCGTAGACAAAATG	TGAGGTCAATGAAGGGGTCGT

Instrumental analysis

A Shimadzu liquid chromatograph (Shimadzu Corporation, Kyoto, Japan) coupling with a QTRAP 5500 mass spectrometer (AB Sciex, Foster) was used to perform the metabolomics analysis. Liquid chromatography was carried out using an amide XBridge high performance liquid chromatographic (HPLC) column (4.6 mm × 100 mm, 3.5 μm, Waters). The mobile phase A was 95% water, 5% acetonitrile, 0.02 M ammonium acetate, and 0.02 M ammonium hydroxide, and mobile phase B was 100% acetonitrile. The gradient program was optimized as follows: 0-3 min, 85% B; 3-12 min, 85-30% B; 12-15 min, 30-2% B; 15-16 min, 2-85% B; 16-23 min, 85% B. The injection volume was 10 μL with a column temperature of 50 ºC. Mass spectrometry date was conducted in positive and negative modes. The temperature of the positive ion source was 475 ºC with 20 to 25 μL min^{-1} nitrogen as the curtain gas. High purity nitrogen was used as the collision gas at a flow rate of 33 μL min^{-1}. The cluster voltage was +93 (positive ion mode), or -93 (negative ion mode). The exit potential of the collision chamber in the positive ion mode was +10, and-10 in the negative ion mode.

For lipidomics, dried samples were reconstituted in acetonitrile/isopropanol/water (65:30:5, v/v/v/) containing 5 mM ammonium acetate. The lipidomics was performed by a Shimadzu liquid chromatography (Shimadzu Corporation, Kyoto, Japan) coupled online to a QTRAP 5500 mass spectrometer (AB Sciex, Foster) operated in multiple reaction monitoring (MRM) mode. The IonSpray voltage was 5500 V in positive mode.

The temperature was 500 °C, the Ion Source Gas 1 (GS1) and Ion Source Gas 2 (GS2) were set as 50. The Collision Gas was set as 'high' and Curtain Gas was 35. In negative mode, the IonSpray voltage was -4500 V, the temperature was 550°C, the other parameters were identical to that in positive mode. A reversed-phase BEH C8 column (2.1 mm × 100 mm, 1.7 μm, Waters) was used for the chromatographic separation of lipids. Mobile phases A were acetonitrile /water (60:40, v/v, 10 mM ammonium acetate) and phases B were isopropanol / acetonitrile (90:10, v/v, 10 mM ammonium acetate). The flow rate was 0.26 mL/min. The column temperature was at 55°C. The elution gradient started with 32% B and was kept for 1.5 min, then linearly increased to 85% B at 15.5 min, and then to 97% B at 15.6 min, and held for 2.4 min. The gradient was back to 32% B at 18.1 min and kept for 1.9 min to equilibrate the column.
